# Randomized Lasso Links Microbial Taxa with Aquatic Functional Groups Inferred from Flow Cytometry

**DOI:** 10.1128/mSystems.00093-19

**Published:** 2019-09-10

**Authors:** Peter Rubbens, Marian L. Schmidt, Ruben Props, Bopaiah A. Biddanda, Nico Boon, Willem Waegeman, Vincent J. Denef

**Affiliations:** a KERMIT, Department of Data Analysis and Mathematical Modelling, Ghent University, Ghent, Belgium; b Department of Ecology and Evolutionary Biology, University of Michigan, Ann Arbor, Michigan, USA; c CMET, Center for Microbial Ecology and Technology, Ghent University, Ghent, Belgium; d Annis Water Resources Institute, Grand Valley State University, Muskegon, Michigan, USA; Florida State University

**Keywords:** 16S rRNA, aquatic microbiology, bacterioplankton, flow cytometry, heterotrophic productivity, machine learning, variable selection

## Abstract

High-nucleic-acid (HNA) and low-nucleic-acid (LNA) bacteria are two operational groups identified by flow cytometry (FCM) in aquatic systems. A number of reports have shown that HNA cell density correlates strongly with heterotrophic production, while LNA cell density does not. However, which taxa are specifically associated with these groups, and by extension, productivity has remained elusive. Here, we addressed this knowledge gap by using a machine learning-based variable selection approach that integrated FCM and 16S rRNA gene sequencing data collected from 14 freshwater lakes spanning a broad range in physicochemical conditions. There was a strong association between bacterial heterotrophic production and HNA absolute cell abundances (*R*^2^ = 0.65), but not with the more abundant LNA cells. This solidifies findings, mainly from marine systems, that HNA and LNA bacteria could be considered separate functional groups, the former contributing a disproportionately large share of carbon cycling. Taxa selected by the models could predict HNA and LNA absolute cell abundances at all taxonomic levels. Selected operational taxonomic units (OTUs) ranged from low to high relative abundance and were mostly lake system specific (89.5% to 99.2%). A subset of selected OTUs was associated with both LNA and HNA groups (12.5% to 33.3%), suggesting either phenotypic plasticity or within-OTU genetic and physiological heterogeneity. These findings may lead to the identification of system-specific putative ecological indicators for heterotrophic productivity. Generally, our approach allows for the association of OTUs with specific functional groups in diverse ecosystems in order to improve our understanding of (microbial) biodiversity-ecosystem functioning relationships.

**IMPORTANCE** A major goal in microbial ecology is to understand how microbial community structure influences ecosystem functioning. Various methods to directly associate bacterial taxa to functional groups in the environment are being developed. In this study, we applied machine learning methods to relate taxonomic data obtained from marker gene surveys to functional groups identified by flow cytometry. This allowed us to identify the taxa that are associated with heterotrophic productivity in freshwater lakes and indicated that the key contributors were highly system specific, regularly rare members of the community, and that some could possibly switch between being low and high contributors. Our approach provides a promising framework to identify taxa that contribute to ecosystem functioning and can be further developed to explore microbial contributions beyond heterotrophic production.

## Abstract (precis)

A major goal in microbial ecology is to understand how microbial community structure influences ecosystem functioning. Various methods to directly associate bacterial taxa to functional groups in the environment are being developed. In this study, we applied machine learning methods to relate taxonomic data obtained from marker gene surveys to functional groups identified by flow cytometry. This allowed us to identify the taxa that are associated with heterotrophic productivity in freshwater lakes and indicated that the key contributors were highly system specific, regularly rare members of the community, and that some could possibly switch between being low and high contributors. Our approach provides a promising framework to identify taxa that contribute to ecosystem functioning and can be further developed to explore microbial contributions beyond heterotrophic production.

## INTRODUCTION

A key goal in the field of microbial ecology is to understand the relationship between microbial diversity and ecosystem functioning. However, it is challenging to associate bacterial taxa to specific ecosystem processes. Marker gene surveys have shown that natural bacterial communities are extremely diverse and that the presence of a taxon does not imply its activity. The taxa observed in these surveys may have low metabolic potential, be dormant, or have recently died (1, 2). An additional hurdle is that the current standard unit of measure for microbial taxonomic analysis is relative abundance. This results in a negative correlation bias (3), which makes it difficult to quantitatively associate specific microbial taxa with microbial ecosystem functions using traditional correlation measures (4). Therefore, in order to ultimately model and predict bacterial communities, new methodologies, which integrate different data types, are needed to associate bacterial taxa with ecosystem functions (5).

One such advance is the use of flow cytometry (FCM), which has been used extensively to study aquatic microbial communities (6–8). This single-cell technology partitions individual microbial cells into phenotypic groups based on their observable optical characteristics. Most commonly, cells are stained with a nucleic acid stain (e.g., SYBR green I) and upon analysis assigned to either a low-nucleic-acid (LNA) or a high-nucleic-acid (HNA) group (9–12). HNA cells differ from LNA cells in both a considerable increase in fluorescence due to cellular nucleic acid content and scatter intensity due to cell morphology. The HNA group is thought to contribute more to productivity of a microbial community, whereas the LNA population has been considered to contribute less (6, 13–15). This is based on positive linear relationships between HNA abundance and (i) bacterial heterotrophic production (BP) (10, 14–17), (ii) bacterial activity measured using the dye 5-cyano-2,3-ditolyl tetrazolium chloride (18, 19), (iii) phytoplankton abundance (20), and (iv) dissolved organic carbon concentrations (21). Additionally, growth rates are higher for HNA cells than for LNA cells (13, 16, 22), and HNA cells accrue cell damage significantly faster than the LNA cells under temperature (23) and chemical oxidant (24) stress. In contrast, LNA bacterial growth rates are positively correlated with temperature and negatively correlated with chlorophyll *a* (25). However, it is important to note that LNA cells are often smaller than HNA cells (9, 12, 25–27), and therefore, LNA cells could have similar amino acid incorporation rates compared to HNA cells when evaluating biomass-specific production (12).

Here we used a data-driven approach to associate the dynamics of individual taxa with those of the LNA and HNA groups in freshwater lakes by adopting a machine learning variable selection strategy. We applied two variable selection methods, the Randomized Lasso (RL) (28) and the Boruta algorithm (29) to associate individual taxa with HNA and LNA cell abundances. These methods extend on traditional machine learning algorithms (i.e., the Lasso and Random forest algorithm for the RL and Boruta algorithm, respectively) by making use of resampling and randomization. These extensions are needed for the following reasons. (i) The Lasso algorithm is not suited for compositional data because the regression coefficients have an unclear interpretation, and single variables may be selected when correlated to other variables (30). (ii) Random Forest algorithms can be biased toward correlated variables (31), which is an intrinsic issue with relative abundance data (3). The extended methods allow the user to either assign a probability of selection (RL) or statistically decide which taxa to select (Boruta).

We generated paired bacterial 16S rRNA gene sequencing and flow cytometry data for 173 samples from three types of lake systems: (i) a set of oligotrophic to eutrophic small inland lakes (62 samples), (ii) a short residence time mesotrophic freshwater estuary lake (Muskegon Lake; 62 samples), and (iii) a large oligotrophic Great Lake (Lake Michigan; 49 samples), all located in Michigan, USA. In addition, we measured bacterial production within 20 of the Muskegon Lake samples using a tritiated-leucine uptake analysis. First, we assessed the correlations between HNA, LNA, and productivity and between individual operational taxonomic units (OTUs) and productivity measurements. Next, we used the RL to associate specific bacterial taxa to HNA and LNA FCM functional groups, and via the observed HNA-productivity relationship, to functioning. We tested whether associated taxa were conserved across lake systems and phylogeny. To validate the RL-based association with the HNA and/or LNA group, we correlated taxon abundances with specific regions within the FCM fingerprint at finer resolution (i.e., bins) without prior knowledge of the HNA or LNA groups. Finally, we performed an additional validation of selected bacterial taxa using the Kendall rank correlation coefficient and the Boruta variable selection algorithm.

## RESULTS

### Study lakes are dominated by LNA cells.

**FIG 1:**
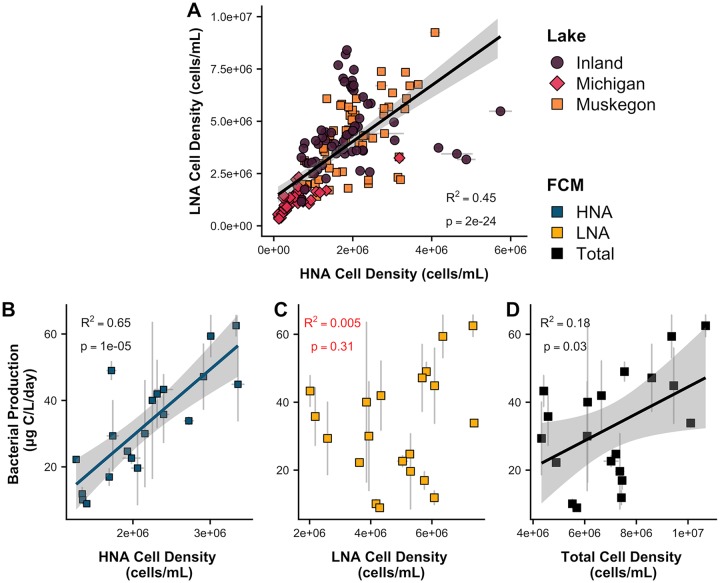
(A) Correlation between HNA cell density (i.e., cell counts) and LNA cell density (i.e., cell counts) across the three freshwater lake ecosystems (173 samples). (B to D) Muskegon Lake bacterial heterotrophic production (20 samples) and its correlation with HNA cell counts (HNAcc) (B), LNA cell counts (LNAcc) (C), and (D) total cell counts. *R*^2^ values represent the adjusted *R*^2^. The gray-shaded areas in the graphs in panels A, B, and D represent the 95% confidence intervals.

The inland lakes (6.3 × 10^6^ cells/ml) and Muskegon Lake (6.0 × 10^6^ cells/ml) had significantly higher total cell abundances than Lake Michigan (1.7 × 10^6^ cell/ml; analysis of variance [ANOVA] *P* = 2.7 × 10^−14^). Across all lakes, the mean proportion of HNA cell counts (HNAcc) to total cell counts was much lower (30.4% ± 9%) compared to the mean proportion of LNA cell counts (LNAcc) (69.6% ± 9%). Through ordinary least-squares regression, there was a strong correlation between HNAcc and LNAcc across all data (adjusted *R*^2^ = 0.45 and *P* = 2 × 10^−24^ **[**Fig. 1A]); however, only Lake Michigan (adjusted *R*^2^ = 0.59, *P* = 5 × 10^−11^) and Muskegon Lake (adjusted *R*^2^ = 0.44, *P* = 2 × 10^−9^) had significant correlations when the three ecosystems were considered separately.

### HNA cell counts and heterotrophic bacterial production are strongly correlated.

For mesotrophic Muskegon Lake, the only lake for which we had heterotrophic production data available, there was a strong correlation between total bacterial heterotrophic production and HNAcc (adjusted *R*^2^ = 0.65 and *P* = 10^−5^ [Fig. 1B]), no correlation between BP and LNAcc (adjusted *R*^2^ = 0.005 and *P* = 0.31 [Fig. 1C]), and a weak correlation between heterotrophic production and total cell counts (adjusted *R*^2^ = 0.18 and *P* = 0.03; [Fig. 1D]). There was a positive (HNA) and negative (LNA) correlation between the fraction of HNA or LNA to total cells and productivity; however, the relationship was weak and not significant (adjusted *R*^2^ = 0.14, *P* = 0.057).

### *Proteobacteria* and OTU481 correlate with productivity measurements.

The Kendall rank correlation coefficient was calculated between centered log ratio (CLR)-transformed abundances of individual OTUs and productivity measurements. An OTU with an unclassified taxonomy, OTU481 was the sole OTU that correlated with productivity after a correction for multiple hypothesis testing (Kendall’s tau-b = −0.67, *P* = 3 × 10^−5^, adjusted *P* = 0.016). At the phylum level, only *Proteobacteria* were significantly correlated to productivity measurements (Kendall’s tau-b = 0.49, *P* = 0.002, adjusted *P* = 0.05).

### Randomized Lasso associates OTUs to HNA and LNA functional groups.

**FIG 2:**
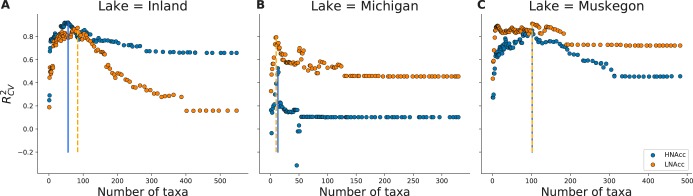
$R_{\text{CV}}^{\text{2}}$ in function of the number of OTUs, which were iteratively removed based on the RL score and evaluated using the Lasso at every step. The solid (HNA) and dashed (LNA) vertical lines correspond to the threshold (i.e., number of OTUs) which resulted in a maximal $R_{\text{CV}}^{\text{2}}$. (A) Inland system; (B) Lake Michigan; (C) Muskegon Lake.

The relevance of specific OTUs for predicting FCM functional group abundance was assessed using the Randomized Lasso (RL), which assigns a score between 0 (i.e., unimportant) to 1 (i.e., very important) to each taxon in function of the target variable: HNAcc or LNAcc. To assess the predictive power of a subset of OTUs based on the RL, we iteratively removed the OTUs with the lowest RL score in a recursive variable elimination scheme.$\;R_{\text{CV}}^{2}$, a goodness-of-fit measure using the *R*^2^ of how well a set of selected OTUs predicts HNAcc or LNAcc compared to true values using cross-validation (CV), increased when lower-ranked OTUs were removed (moving from right to left on Fig. 2). The increase was gradual for the inland lakes (Fig. 2A) and Muskegon Lake (Fig. 2C) but was abrupt for Lake Michigan (Fig. 2B). The proportion of taxa that resulted in the highest $R_{\text{CV}}^{\text{2}}$ (see solid [HNA] and dotted [LNA] lines in Fig. 2) was 10.2% of all taxa for HNA and 17.7% for LNA for the inland lakes, 4.0% for HNA and 3.0% for LNA for Lake Michigan, and 21.1% for both HNA and LNA in Muskegon Lake. To test the robustness of the procedure, it was evaluated using independent test sets with a nested cross-validation (NCV) scheme, denoted as $R_{\text{NCV}}^{2}$ (i.e., in the outer loop, samples are split from the data set to create a test set, and in the inner loop, the RL is applied and the Lasso is fitted and optimized). Muskegon Lake resulted in the highest $R_{\text{NCV}}^{2}$ (HNAcc, 0.49; LNAcc, 0.65), followed by Lake Michigan (HNAcc, 0.41; LNAcc, 0.34), and the inland lakes (HNAcc, 0.40; LNAcc, 0.31). As the $R_{\text{NCV}}^{\text{2}}$ value is considerably higher than zero, selected subsets of OTUs are considered to be predictive of changes in HNAcc and LNAcc for unseen samples. No relationship could be established between the RL score and the relative abundance of individual OTUs (see Fig. S1 at https://doi.org/10.6084/m9.figshare.8218775.v3). HNAcc and LNAcc could be predicted with equivalent performance to relative HNA and LNA proportions, yet the increase between initial and optimal performance was larger according to the $R_{\text{CV}}^{\text{2}}$ (see Fig. S2 at https://doi.org/10.6084/m9.figshare.8218775.v3). The $R_{\text{CV}}^{\text{2}}$ was higher when relative OTU abundances were transformed using the CLR transformation (see Fig. S3 at https://doi.org/10.6084/m9.figshare.8218775.v3). OTU481 had a low RL score (0.022) for HNAcc. Of the top 10 OTUs selected for HNAcc according to the RL, three were significantly associated with productivity (OTU614: *P* = 0.0064; OTU412, *P* = 0.044; OTU487, *P* = 0.014), but not when corrected for multiple hypothesis testing.

### HNA and LNA RL-associated OTUs differed across lake systems.

**FIG 3:**
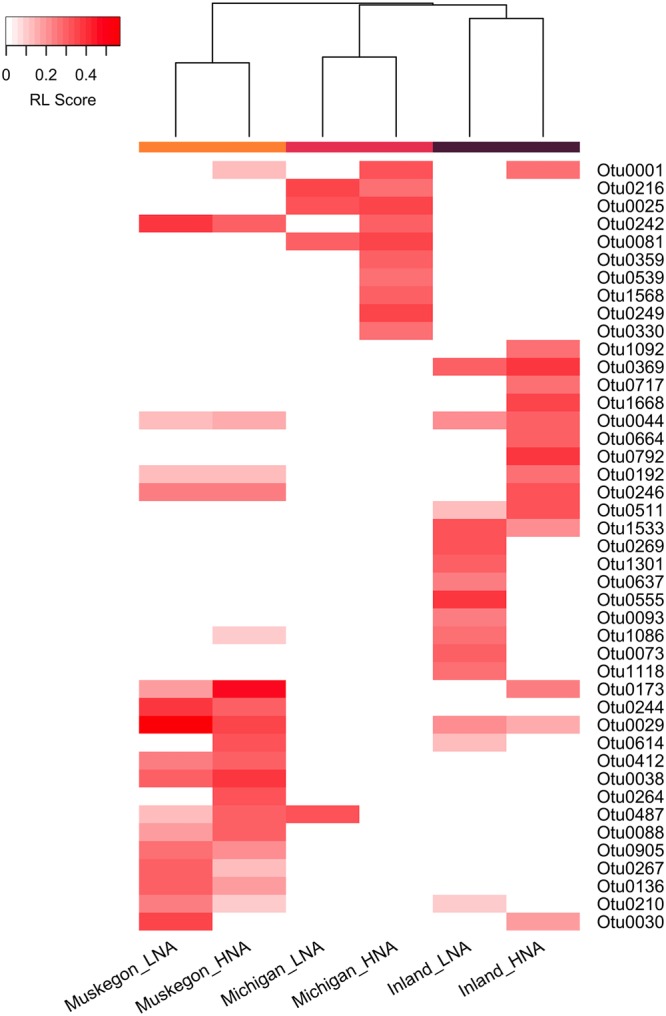
Hierarchical clustering of the RL score for the top 10 selected OTUs within each lake system and flow cytometry functional groups with the selected OTUs (rows) across HNA and LNA groups within the three lake systems (columns). Column header colors: Muskegon Lake, orange; Lake Michigan, pink; inland lakes, purple.

RL-selected OTUs were mostly assigned to either the HNA or LNA group, and there was limited correspondence across lake systems between the selected OTUs (Fig. 3). Of the OTUs selected for Lake Michigan, 1.5% to 1.9% were also associated with HNAcc or LNAcc for the inland lakes or Muskegon Lake. This amount was higher for the shared OTUs between the inland lakes and Muskegon Lake, but it still amounted to only 6.0% (HNAcc) or 10.5% (LNAcc) of all common OTUs. For OTUs selected in all three freshwater environments, RL scores were lake system specific, with only a significant similarity between the inland lakes and Muskegon Lake for HNAcc (Pearson's *r* = 0.21 and *P* = 0.0042 [see Fig. S4 at https://doi.org/10.6084/m9.figshare.8218775.v3]).

**FIG 4:**
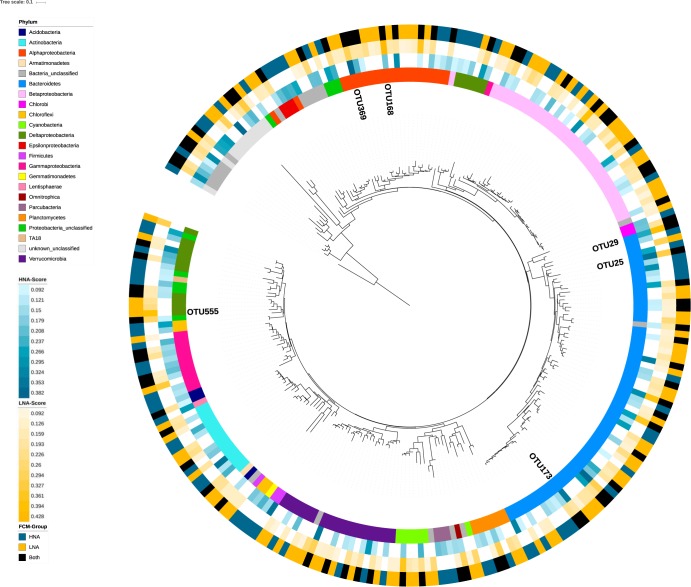
16S rRNA gene phylogenetic tree with all HNA and LNA selected OTUs from each of the three lake systems with their taxonomic classification, RL scores, and association. These factors are shown starting from the inside working to the outside as follows: (i) phylum-level taxonomic classification, (ii) HNA RL scores (i.e., HNA-Score), (iii) LNA RL scores (i.e., LNA-Score), and (iv) discrete association with HNA, LNA, or both groups based on the RL score threshold values (i.e., FCM-Group). Any OTU absent from a FCM group is white. The tree was rooted using OTU1552.

We constructed a phylogenetic tree in function of the RL score (Fig. 4). The *Bacteroidetes*, *Betaproteobacteria*, *Alphaproteobacteria*, and *Verrucomicrobia* contributed 54% of the 258 OTUs selected by the RL. Most selected OTUs belonging to these four phyla were associated with the LNA group (41 to 52% of selected OTUs), less than one third with the HNA group (14 to 30% of selected OTUs), and the remainder were selected as associated with both the LNA and HNA groups (23 to 36% of selected OTUs). In Muskegon Lake, OTU173 (*Bacteroidetes*;*Flavobacteriales*;bacII-A) was selected as the major HNA-associated taxon, while OTU29 (*Bacteroidetes*;*Cytophagales*;bacIII-B) had the highest RL score for LNA OTUs. In Lake Michigan, OTU25 (*Bacteroidetes;Cytophagales*;bacIII-A) was selected as the major HNA-associated taxon, while OTU168 (*Alphaproteobacteria*:*Rhizobiales*:alfVII) was selected as a major LNA-associated taxon. For the inland lakes, OTU369 (*Alphaproteobacteria*;*Rhodospirillales*;alfVIII) was the major HNA-associated OTU, while OTU555 (Deltaproteobacteria;*Bdellovibrionaceae*;OM27) was the major LNA-associated taxon. Most OTUs were selected for Muskegon Lake (153 OTUs; compared to 136 OTUs from the inland lakes and 20 OTUs from Lake Michigan), and 33% of these OTUs were associated with both FCM groups, including all of the top OTUs except for OTU555.

### HNA and LNA functional groups display no phylogenetic signal.

A recent study inferred phylum-level phylogenetic resolution of large-cell (i.e., HNA) and small-cell (i.e., LNA) taxa (27) from five distinct freshwater systems, indicating that these FCM groups are deeply rooted phylogenetic traits. However, samples from freshwater lakes in their data set often had multiple (rather than two) FCM groups, suggesting the hypothesis that freshwater lake bacterial taxa are less likely to be phylogenetically conserved. Thus, we sought to test whether phylogenetically related OTUs in our data set also resembled each other in HNA and LNA association. To evaluate how much evolutionary history explains whether a selected taxon was associated with the HNA and/or LNA group(s), we calculated Pagel’s λ, Blomberg’s K, and Moran’s I for testing whether there was a phylogenetic signal of these traits based on the phylogenetic tree in Fig. 4. No phylogenetic signal was detected when using Pagel’s λ with FCM functional group as a discrete variable (i.e., associating an OTU with HNA, LNA, or both) or in relation to the HNA RL score, where the RL score represents a continuous variable (lambda = 0.16; *P* = 1). However, there was a significant phylogenetic signal for the LNA RL score (*P* = 0.003, λ = 0.66), suggesting a stronger phylogenetic structure in the LNA group than in the HNA group. Though, this significant result in the LNA group was not found when other measures of phylogenetic signal were considered (Blomberg’s K (HNA, *P* = 0.63; LNA, *P* = 0.54), and Moran’s I (HNA, *P* = 0.88; LNA, *P* = 0.12).

We applied the RL to all other taxonomic levels (see Fig. S5A to C at https://doi.org/10.6084/m9.figshare.8218775.v3). RL scores increased as less resolved taxonomic levels were considered (i.e., highest for phylum, lowest for OTU) (see Fig. S5A to C at https://doi.org/10.6084/m9.figshare.8218775.v3). The $R_{\text{NCV}}^{2}$ at the phylum, order, and genus level indicated that our results were consistent across all taxonomic levels and that different levels of phylogeny can be related to changes in HNAcc and LNAcc (see Fig. S5D at https://doi.org/10.6084/m9.figshare.8218775.v3). The fraction of variables (i.e., taxa) that could be removed to reach the maximum $R_{\text{CV}}^{2}$ decreased as the taxonomic level became less resolved (see Fig. S5E at https://doi.org/10.6084/m9.figshare.8218775.v3). In general, these results show that the proposed methodology is applicable to different levels of taxonomy but motivates the absence of a phylogenetic signal in the HNA and LNA group.

### Top-ranked taxa are highly correlated with specific subregions in the FCM fingerprint that respect the HNA and LNA dichotomy.

**FIG 5:**
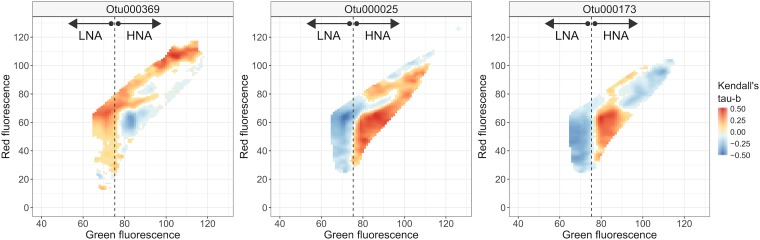
Correlation (Kendall’s tau-b) between the relative abundances and the densities inside each bin in the cytometric fingerprint for the top-ranked OTU in each lake system according to the RL. The fluorescence threshold used to manually define HNA and LNA populations is indicated by the dashed line. Results for inland lakes (left), Lake Michigan (middle), and Muskegon Lake (right) are shown.

To confirm the association of the final selected OTUs with the HNA and LNA groups, we resolved how HNA and LNA groups correspond to OTU-level clustering of cells in the FCM fingerprints. We calculated the correlation between the density of individual small regions (i.e., “bins”) in the flow cytometry data with the relative abundances of the top-ranked OTUs according to the RL (see Table 1). Please note the following: (i) As these values denote correlations, they do not indicate actual presence. (ii) The threshold that was used to manually make the distinction between HNAcc and LNAcc (i.e., dashed line in Fig. 5) lies very close to the border between the two regions of positive and negative correlation. OTU25 correlated with bins that when aggregated corresponded to almost the entire HNA region, whereas OTU173 was limited to bins corresponding to the bottom of the HNA region (Fig. 5). In contrast, OTU369 was positively correlated to bins situated in both the LNA and HNA regions of the cytometric fingerprint, highlighting results from Fig. 3 and Fig. 4 where OTU369 was selected for both HNA and LNA.

### Validation of RL OTU selection results using the Boruta algorithm and Kendall’s tau-b.

Associations between OTUs and HNAcc and LNAcc were additionally investigated using Kendall’s tau-b and the Boruta variable selection algorithm (an algorithm that statistically selects relevant variables based on the importance of the permuted compared to original variables as retrieved from multiple Random Forest models). Venn diagrams were constructed to visualize consistency in the number of OTUs that were selected according to all methods, including the RL (see Fig. S6 at https://doi.org/10.6084/m9.figshare.8218775.v3). All methods agreed on including only a small subset of OTUs for the best model. The Kendall rank correlation coefficient selected the most OTUs, followed by the RL, and then the Boruta algorithm (except for HNAcc in Lake Muskegon [see Fig. S6 at https://doi.org/10.6084/m9.figshare.8218775.v3]).

For each lake system individually, the top RL-scored OTU for HNAcc was also selected by the Boruta algorithm, whereas both methods agreed only for the top-ranked OTU in Lake Michigan for LNAcc (Table 1). Across all lake systems, most selected OTUs were lake system specific (see Fig. S7 at https://doi.org/10.6084/m9.figshare.8218775.v3). Only OTU060 (*Proteobacteria*;*Sphingomonadales*;alfIV_unclassified) was selected across all lake systems (LNAcc associated). The subset selected by the Boruta algorithm, in combination with Random Forest predictions, resulted in a lower $R_{\text{CV}}^{2}$ compared to Lasso predictions based on the RL score (see Fig. S8 at https://doi.org/10.6084/m9.figshare.8218775.v3).

**TABLE 1:** Top-ranked OTUs according to the Randomized Lasso (RL) per flow cytometry functional group and lake ecosystem^*a*^

Lake system and functional group	OTU	RL score	Boruta selected	HNA		LNA		Phylum	Class	Order	Family	Genus (species)
				Kendall’s tau-b	*P* value	Kendall’s tau-b	*P* value					
Inland												
HNA	OTU369	0.382	Yes	−0.43	<0.001	−0.28	7.9 × 10^−7^	*Proteobacteria*	*Alphaproteobacteria*	*Rhodospirillales*	alfVIII	alfVIII_unclassified
LNA	OTU555	0.384	No	0.089	NS	0.22	0.011	*Proteobacteria*	Deltaproteobacteria	*Bdellovibrionales*	*Bdellovibrionaceae*	OM27_clade

Michigan												
HNA	OTU025	0.362	Yes	0.46	<0.001	0.41	<3.5 × 10^−6^	*Bacteroidetes*	*Cytophagia*	*Cytophagales*	bacIII	bacIII-A
LNA	OTU168	0.428	Yes	0.26	0.0092	0.4	5.9 × 10^−5^	*Proteobacteria*	*Alphaproteobacteria*	*Rhizobiales*	alfVII	alfVII_unclassified

Muskegon												
HNA	OTU173	0.462	Yes	0.5	<0.001	0.2	8.2 × 10^−9^	*Bacteroidetes*	*Flavobacteriia*	*Flavobacteriales*	bacII	bacII-A
LNA	OTU029	0.568	No	0.26	0.0029	0.49	1.4 × 10^−8^	*Bacteroidetes*	*Cytophagia*	*Cytophagales*	bacIII	bacIII-B (Algor)

a Selection according to the Boruta algorithm is given in addition to the RL score. Descriptive statistics by means of the Kendall rank correlation coefficient have been added with the level of significance in the function of the HNA or LNA group (NS, not significant).

Although all methods agreed only on a minority of OTUs, the results are consistent in multiple ways, which allow us to formulate a number of more general conclusions across these methods. (i) The selected OTUs were mostly lake system specific. (ii) Small fractions of OTUs allow us to predict changes in community composition. (iii) Selected OTUs were associated with absolute HNA or LNA abundance. (iv) Top RL-ranked HNA-associated OTUs were also selected according to the Boruta algorithm. (v) When the RL and Boruta both agreed on an OTU, it was always significantly correlated with either HNAcc or LNAcc.

## DISCUSSION

Our study furthers the integration of functional and genotypic information to determine the complex relationships between microbial diversity and ecosystem functioning. Our results confirmed previous findings that flow cytometric (FCM) operational groups are distinct functional groups having divergent correlations with heterotrophic productivity. Using two machine learning-based variable selection strategies, we associated bacterial taxa identified by 16S rRNA gene sequencing to two functional groups in three types of freshwater lake systems in the Great Lakes region. We revealed the following: (i) HNA and LNA cell abundances could be predicted by a small subset of OTUs that were unique to each lake type. (ii) Some OTUs were included in the best model for both HNA and LNA abundance. (iii) There was no phylogenetic conservation of HNA and LNA group association. (iv) Although the correlations between individual OTUs and FCM data support the dichotomy of HNA and LNA, variation in OTU relative abundance correlated best with shifts in cell numbers in smaller subregions of the FCM fingerprint and not the entire HNA or LNA region.

Only the association between bacterial heterotrophic production (BP) and HNAcc was strong and significant. While many studies have reported no association between HNA and bacterial production, our result is in line with some previous reports (9, 14–16). However, past studies have focused on the proportion of HNA rather than absolute cell abundances. For example, Bouvier et al. (11) found a correlation between the fraction of HNA cells and BP within a large data set of 640 samples across various freshwater to marine environments (Pearson’s *r* = 0.49), whereas a study off the coast of the Antarctic Peninsula found a moderate correlation (*R*^2^ = 0.36) (17). Another study in the Bay of Biscay also found this association (*R*^2^ = 0.16) (15); however, the authors attributed this difference to be related to cell size and not due to the activity of HNA. Notably, these studies were predominantly testing the association of marine HNA groups. The high correlation coefficients observed in our study may indicate a strong coupling between freshwater carbon cycling and HNA group abundance in freshwater lake systems. Consequently, this suggests an important contribution of HNA bacteria in the disproportionately large role that freshwater systems play in the global carbon cycle (32). It has to be noted that our study evaluated bacterial heterotrophic production using leucine amino acid incorporation, which biases our analyses against bacterial groups that cannot import or assimilate this compound (33). HNA cells have significantly higher incorporation rates of both leucine (as used in this study) and thymidine (34). Finally, as our correlations with proportional HNA group abundances also indicated less strong correlations than with absolute HNAcc, we suggest that absolute HNAcc should be used to best predict and study heterotrophic bacterial production.

Similar to other microbiome studies that use machine learning, only a minority of OTUs were needed to predict the phenotype of interest, with low predictive power of each single OTU, but strong predictive capacity of the selected group of OTUs (17, 35–37). Both the RL and Boruta algorithm have been applied to microbiome studies before, for example in the selection of genera in the human microbiome associated with body mass index (BMI) (38), salivary pH and lysozyme activity (39), and in relation to multiple sclerosis (40) or with differing diets during primate pregnancy (41). The Boruta algorithm has also recently been proposed as one of the top-performing variable selection methods that make use of Random Forests (42). Despite the power of these approaches, improvements can be made when attempting to integrate different types of data. For example, 16S rRNA gene sequencing still faces the hurdles of DNA extraction (43) and 16S copy number bias (44). Moreover, detection limits are different for FCM (expressed in the number of cells) and 16S rRNA gene sequencing (expressed in the number of gene counts or relative abundance), therefore creating an issue that data may be different in resolution.

The selection of different sets of HNA and LNA OTUs across the three freshwater systems indicates that different taxa underlie the universally observed HNA and LNA functional groups across aquatic systems. This is perhaps not surprising, as it has been shown that there is strong species sorting in lake systems (45, 46), shaping community composition through diverging environmental conditions between the lake systems presented here (47). This high system specificity also explains the low RL scores for individual OTUs, as the spatial and temporal dynamics of an OTU diverged strongly across systems. For example, an OTU that has an RL score of 0.5 implies that on average, it will be chosen only one out of two times in a Lasso model.

Some OTUs were associated with both HNAcc and LNAcc. There are multiple possible explanations for this. (i) In line with scenario 1 from Bouvier et al. (11), cells transition from active growth (primarily HNA) to death or a dormant state (primarily LNA), depending on variable conditions over the spatiotemporal gradients sampled in this study. A large fraction of cells (40 to 95%) in aquatic systems has indeed been inferred to be dormant (48–50), in line with the predominance of LNA cells. (ii) The same OTU may occur in both HNA and LNA groups due to phenotypic plasticity, which is more in line with scenario 4 from Bouvier et al. (11). Bacterial phenotypic plasticity in size and morphology has been observed (51) and agrees with suggestions that HNA and LNA groups correspond to cells of different sizes (12, 15, 27). (iii) The association of taxa to LNA and HNA can also mean that these taxonomic groups thrive within either high- or low-productivity ecosystems and not necessarily that they are responsible for the change in productivity. (iv) Finally, OTU-level grouping of bacterial taxa can disguise genomic and corresponding phenotypic heterogeneity (52–55), which may be an alternate explanation for inconsistent associations between OTUs and FCM functional groups.

We found no clear phylogenetic signal of HNAcc or LNAcc association. This agrees with the freshwater lake samples collected by Proctor et al. (27) that sometimes had multiple FCM groups rather than the typical two for HNA and LNA groups. However, it is in contrast to the clear phylum-level signal of small LNA and large HNA taxa across different aquatic systems (27), suggesting that this is a deeply rooted and conserved evolutionary trait, rather than a transient physiological trait, in the bacterial phylogenetic tree. In addition, it is notable that Proctor et al. (27) separated HNA and LNA cells based on cell size. HNA cells were defined at approximately >0.4 μm and LNA cells were approximately 0.2 to 0.4 μm, based on 50 to 90% removal of HNA cells after filtering using a 0.4-μm filter. Our study instead separated these FCM functional groups on the traditional basis of fluorescence intensity alone. A more direct estimation of phylogenetic conservation that directly combines cell sorting of HNA or LNA cells and sequencing, such as the approach of Vila-Costa et al. (56), will be needed to resolve these contrasting results. Considering the correlations between FCM-based phenotypic diversity and sequencing-based taxonomic diversity (57, 58), there is clearly a link between taxonomy and the structure in microbial flow cytometry data (17). However, the HNA and LNA dichotomy may be unresolved, as a number of reports have identified more than two FCM operational groups in aquatic systems (17, 27, 56, 59, 60). This is in line with our correlation analysis revealing that highly ranked OTUs are connected to specific subregions in the FCM fingerprint that respect a general HNA/LNA structure (Fig. 5).

The Boruta algorithm and RL scores agreed on a small subset of OTUs, including the top-ranked HNA OTU for all lake systems according to RL, which motivates further investigation of the ecology of these OTUs. While little detailed information on the identities and ecology of HNA and LNA freshwater lake bacterial taxa exists, several studies identified *Bacteroidetes* among the most prominent HNA taxa, which is in line with our findings. Independent research by Vila-Costa et al. (56) found that the HNA group was dominated by *Bacteroidetes* in summer samples from the Mediterranean Sea, Read et al. (19) showed that HNA abundances correlated with *Bacteroidetes*, and Schattenhofer et al. (61) reported that the *Bacteroidetes* accounted for the majority of HNA cells in the North Atlantic Ocean. In Muskegon Lake, OTU173 was the dominant HNA taxon and is a member of the order *Flavobacteriales* (bacII-A). The bacII group is a very abundant freshwater bacterial group and has been associated with senescence and decline of an intense algal bloom (62), suggesting their potential for bacterial production. The bacII-A group has also made up ∼10% of the total microbial community during cyanobacterial blooms, reaching its maximum density immediately after a bloom (63). In Lake Michigan, OTU25, a member of the *Bacteroidetes* order *Cytophagales* known as bacIII-A, was the top HNA OTU. However, much less is known about this specific group of *Bacteroidetes*. The bacII-A/bacIII-A group has been strongly associated with more heterotrophically productive headwater sites (compared to higher-order streams) from the River Thames, showing a negative correlation in rivers with dendritic distance from the headwaters, indicating that these taxa may contribute more to productivity (19). In the inland lakes, OTU369 was the major HNA taxon and is associated with the *Alphaproteobacteria* order *Rhodospirillales* (alfVIII), which to our knowledge is a group with little information available in the literature. In contrast to our findings of *Bacteroidetes* and *Alphaproteobacteria* HNA selected OTUs, Tada and Suzuki (64) found that the major HNA taxon from an oceanic algal culture was from the *Betaproteobacteria*, whereas LNA OTUs were within the *Actinobacteria* phylum.

### Conclusions.

We integrated flow cytometry and 16S rRNA gene amplicon sequencing data to associate bacterial taxa with productivity in freshwater lake systems. Our results on a diverse set of freshwater lake systems indicate that the taxa associated with HNA and LNA functional groups are lake specific and that association with these functional groups is not phylogenetically conserved. With this study, we show the potential and limitations of integrating flow cytometry-derived *in situ* functional information with sequencing data using machine learning approaches. This integration of data enhances our insights into which taxa may contribute to ecosystem functioning in aquatic bacterial communities. While these data-driven hypotheses will need further verification, the method is promising considering the wide application of FCM in aquatic environments, its recent application in other sample matrices (e.g., feces [65], soils [66], and wastewater sludge [67]), and the introduction of novel stains to delineate operational groups based on phenotypic traits (68).

## MATERIALS AND METHODS

### Data collection and DNA extraction, sequencing, and processing.

In this study, we used a total of 173 samples collected from three types of lake systems described previously (47), including: (i) 49 samples from Lake Michigan (2013 and 2015), (ii) 62 samples from Muskegon Lake (2013 to 2015; one of Lake Michigan’s estuaries), and (iii) 62 samples from 12 inland lakes in southeastern Michigan (2014 to 2015). For more details on sampling, please see Fig. 1 and the “Field Sampling,” “DNA extraction,” and “DNA sequencing and processing” sections in Chiang et al. (47). In all cases, water for microbial biomass samples was collected and poured through a 210-μm and 20-μm bleach-sterilized nitex mesh, and sequential in-line filtration was performed using 47-mm polycarbonate in-line filter holders (Pall Corporation, Ann Arbor, MI, USA) and an E/S portable peristaltic pump with an easy-load L/S pump head (Masterflex; Cole Parmer Instrument Company, Vernon Hills, IL, USA) to filter first through a 3-μm isopore polycarbonate (TSTP, 47-mm diameter; Millipore, Billerica, MA, USA) and second through a 0.22-μm Express Plus polyethersulfone membrane filter (47-mm diameter; Millipore, MA, USA). The current study utilized only the 3- to 0.22-μm fraction for analyses.

DNA extractions and sequencing were performed as described in Chiang et al. (47). Briefly, DNA extractions were performed using a modified AllPrep DNA/RNA kit (Qiagen, Venlo, The Netherlands) (43). Sequencing was performed at the University of Michigan Medical School on an Illumina MiSeq platform with v2 chemistry 2 × 250 (500 cycles) using dual index-labeled primers that target the V4 region of the 16S rRNA gene (515F/806R) (69). Fastq files were submitted to the National Center for Biotechnology Information (NCBI) sequence read archive under BioProject accession number PRJNA414423 (inland lakes), PRJNA412983 (Lake Michigan), and PRJNA412984 (Muskegon Lake). We analyzed the sequence data using MOTHUR V.1.38.0 (seed = 777) (70) based on the MiSeq standard operating procedure and put together at the following link: https://github.com/rprops/Mothur_oligo_batch. A combination of the Silva Database (release 123) (71) and the freshwater TaxAss 16S rRNA database and pipeline (72) was used for classification of operational taxonomic units (OTUs).

For the taxonomic analysis, each of the three lake data sets were analyzed separately with an OTU abundance threshold cutoff of at least five sequences in 10% of the samples in the data set (similar strategy to the strategy in reference 73). For comparison of taxonomic abundances across samples, each of the three data sets were then rarefied to an even sequencing depth, which was 4,491 sequences for Muskegon Lake samples, 5,724 sequences for the Lake Michigan samples, and 9,037 sequences for the inland lake samples. Next, the relative abundance at the OTU level was calculated using the *transform_sample_counts()* function in the phyloseq R package (74) by taking the count value and dividing it by the sequencing depth of the sample. For all other taxonomic levels, the taxonomy was merged at certain taxonomic ranks using the *tax_glom()* function in phyloseq (74), and the relative abundance was recalculated.

### Heterotrophic bacterial production measurements.

Muskegon Lake samples from 2014 and 2015 were processed for heterotrophic bacterial production using the [^3^H]leucine incorporation into bacterial protein in the dark method (75, 76). At the end of the incubation with [^3^H]leucine, cold trichloroacetic acid-extracted samples were filtered onto 0.2-μm filters that represented the leucine incorporation by the bacterial community. Measured leucine incorporation during the incubation was converted to bacterial carbon production rate using a standard theoretical conversion factor of 2.3 kg of C per mole of leucine (76).

### Flow cytometry, measuring HNA and LNA.

In the field, a total of 1 ml of 20-μm-filtered lake water was fixed with 5 μl of glutaraldehyde (20% [vol/vol] stock), incubated for 10 min on the bench (covered with aluminum foil to protect from light degradation), and then flash frozen in liquid nitrogen to be stored later in a freezer at –80°C until processing with a flow cytometer. Flow cytometry (FCM) procedures followed the protocol laid out by Props et al. (57), which also uses some of the samples presented in the current study (i.e., Lake Michigan and Muskegon Lake samples). Samples were thawed and stained with SYBR green I to a final concentration of 1× SYBR green I and measured in triplicate. After incubation for 20 min at 37°C in the dark, the samples were analyzed on a BD Accuri C6 cytometer (BD Biosciences, Erembodegem, Belgium) in fixed-volume mode. The resulting multiparameter data were then analyzed in the following ways. First, a fixed single gate is used to separate bacterial cells from background noise for all samples using the green (FL1-H; 530/30-nm) versus red (FL3-H; >670-nm) fluorescence detectors. The lowest number of cells collected after denoising was 2,342. Next, HNA and LNA groups were selected by applying two fixed gates to all samples using the same detectors as introduced by Prest et al. (77) and plotted in Fig. S9 at https://doi.org/10.6084/m9.figshare.8218775.v3. Therefore, the same threshold was used for all samples to distinguish HNA cells from LNA cells using the green and red fluorescence channels. Cell counts were determined per HNA and LNA group and averaged over the three replicates (giving rise to HNAcc and LNAcc, with units of cells per ml). All cytometry data are available in the FlowRepository database (78): inland lakes (ID:FR-FCM-ZY9J), Lake Michigan and Muskegon Lake (ID:FR-FCM-ZYZN).

### Data analysis.

**(i) FCM statistics.** We tested the difference in absolute number of cells within HNA and LNA functional groups by running analysis of variance with a *posthoc* Tukey’s honestly significant difference (HSD) test [*aov()* and *TukeyHSD()*; *stats* R package] (76). In addition, we tested the association of HNA and LNA to each other by running ordinary least-squares regression with the *lm()* function (*stats* R package) (79).

**(ii) FCM productivity statistics.** The association of HNA and LNA cell counts (HNAcc/LNAcc) with productivity was tested by running ordinary least-squares regression with the *lm()* function (*stats* R package) (79). This was also done for the total cell counts and relative fraction of HNA cell counts (by dividing HNAcc by the total cell counts).

**(iii) 16S rRNA gene sequencing productivity statistics.** The Kendall ranking correlation coefficient or Kendall’s tau-b between productivity measurements and individual abundances of taxa were calculated on the phylum and OTU level using the *kendalltau()* function from Scipy (v1.0.0). The “tau-b” implementation was used, which is able to deal with ties. Values range from −1 (i.e., strong disagreement) to 1 (i.e., strong agreement). *P* values were corrected using Benjamini-Hochberg correction, reported as adjusted *P* values. This was done using the *multitest()* function from the Python module Statsmodels (80) (v0.5.0).

**(iv) RL associations between 16S rRNA gene sequencing and FCM functional groups.** Taxa were associated with functional measurements through FCM by using the Randomized Lasso (RL) (28). However, before applying the method, the data first were preprocessed following the guidelines of Paliy and Shanker (81), Gloor et al. (3), and Quinn et al. (82). The relative abundances of OTUs were transformed using a centered log ratio (CLR) transformation before variable selection was applied. This means that the relative abundance *x_i_* of a taxon was transformed according to the geometric mean of that sample, in which there are *p* taxa present:

$${x_{i}}^{\prime }=\log\left(x_{i}/{\left(\overset{p}{\underset{j=1}{\prod }}x_{j}\right)}^{1/p}\right)$$

Zero values were replaced by δ = 1/*p*^2^. This was done using the scikit-bio package (www.scikit-bio.org, v0.4.1).

The RL is based on an extension of the Lasso estimator. In the case of *n* samples, the Lasso estimator fits the following regression model:

β^λ=arg min⁡β∈ℝp ‖y−Xβ‖22+λ∑j=1p|βj|

in which *X* denotes the abundance table, *y* is the target to predict, which is either HNA cell abundances (HNAcc) or LNA cell abundances (LNAcc), β is the weight of each variable and λ is a regularization parameter that controls the complexity of the model and prevents overfitting. The Lasso performs an intrinsic form of variable selection, as the weights of certain variables will be set at zero.

Stability selection, when applied to the Lasso, is in essence an extension of the Lasso regression. It implements two types of randomizations to assign a score to the variables, and is therefore also called the Randomized Lasso. The resulting RL score can be seen as the probability that a certain variable will be included in a Lasso regression model (i.e., its weight will be nonzero when fitted). When performing stability selection, the Lasso is fitted to *B* different subsamples of the data of fraction *n*/2, denoted as *X*′ and corresponding *y*′. A second randomization is added by introducing a weakness parameter α. In each model, the penalty λ changes to a randomly chosen value in the set [λ, λ/*α*], which means that a higher penalty will be assigned to a random subset of the total amount of variables. The Randomized Lasso therefore becomes

β^λ=arg min⁡β∈ℝp ‖y′−X′β‖22+λ∑j=1p|βj|wj

where *w_j_* is a random variable which is either α or 1. Next, the Randomized Lasso score (RL score) is determined by counting the number of times the weight of a variable was not zero for each of the *B* models and divided by *B*. Meinshausen and Bühlmann (28) show that, under stringent conditions, the number of falsely selected variables is controlled for the Randomized Lasso when the RL score is higher than 0.5. If λ is varied, one can determine the stability path, which is the relationship between the RL score and λ for every variable. For our implementation, *B * = * *500, *α* = 0.5, and the highest score was selected in the stability path for which λ ranged from 10^−3^ until 10^3^, logarithmically divided in 100 intervals. The *RandomizedLasso()* function from the scikit-learn machine learning library was used (83) (v0.19.1).

A recursive variable elimination strategy was applied to evaluate the predictive power of scores assigned by the RL (84). Variables were ranked according to the RL score. Next, the lowest-ranked variables were eliminated from the data set, after which the Lasso was applied to predict HNAcc and LNAcc, respectively. This process was repeated until only the highest-scored taxa remained. In this way, performance of the Randomized Lasso was assessed from a minimal-optimal evaluation perspective (85). This means that the lowest number of variables was determined that resulted in the highest predictive performance.

In order to account for the spatiotemporal structure of the data, a blocked cross-validation scheme was implemented (86). Samples were grouped according the site and year that they were collected. This results in 5, 10, and 16 distinctive groups for the Lake Michigan, Muskegon Lake, and inland lake systems, respectively. Predictive models were optimized in function of the *R*^2^ between predicted and true values of held-out groups using a leave-one-group-out cross-validation scheme with the *LeaveOneGroupOut()* function. This results in a cross-validated $R_{\text{CV}}^{2}$ value. For the Lasso, λ was determined using the *lassoCV()* function, with setting eps = 10^−4^ and n_alphas = 400. All functions are part of scikit-learn (83) (v0.19.1). In order to test the generalizability of the procedure, a nested leave-one-group-out cross-validation procedure was implemented as well. First, samples are split from the data set to create a test set, and in the inner loop, the RL is applied and the Lasso is fitted and optimized. Predictions for the different test sets were concatenated, evaluated, and summarized, denoted as $R_{\text{NCV}}^{\text{2}}$.

**(v) Associations between 16S rRNA gene sequencing and FCM across ecosystems.** To visualize patterns of the top 10 RL-selected HNA and LNA OTUs across the three ecosystems, a heatmap was created with the RL scores of each OTU from the Randomized Lasso regression that were higher than specified threshold values. The heatmap was created with the *heatmap.2()* function (*gplots* R package) using the Euclidean distances of the RL scores and a complete linkage hierarchical clustering algorithm (Fig. 3). Similarity of RL scores between lake systems and functional groups was quantified using the Pearson correlation. This was done using the *pearsonr()* function in Scipy (v1.0.0).

**(vi) Associations between 16S rRNA gene sequencing and FCM across phylogeny.** To assess the influence of phylogeny, abundances of taxa were determined at all additional taxonomic levels by merging taxa at the OTU level according to their shared taxonomic level. The same procedure as reported in “RL associations between 16S rRNA gene sequencing and FCM functional groups” above was then applied at the phylum, order, and genus level in the function of HNAcc and LNAcc.

We calculated the best performing maximum likelihood phylogenetic tree using the GTR-CAT model (-gtr -fastest) model of nucleotide substitution with FastTree (version 2.1.9 No SSE3) (87) and visualized using the interactive tree of life (iTOL) (88) in Fig. 4. Phylogenetic signal is a measure of the dependence among a species’ trait values on their phylogenetic history (89). If the phylogenetic signal is very strong, taxa belonging to similar phylogenetic groups (e.g., a phylum) will share the same trait (i.e., association with HNAcc or LNAcc). Alternatively, if the phylogenetic signal is weak, taxa within a similar phylogenetic group will have different traits. The phylogenetic signal was measured with both discrete (i.e., HNA, LNA, or both) and continuous (i.e., the RL score) traits using the newick tree from FastTree. For the most part, Pagel’s lambda was used (90) to test for phylogenetic signal and was calculated with the *fitDiscrete()* function from the geiger R package (discrete trait) (91) and the *phylosig()* function from the phytools R package (continuous trait) (92). The lambda value varies between 0 and 1, with 1 indicating complete phylogenetic patterning and 0 representing no phylogenetic patterning, leading to a tree collapsing into a single polytomy. In addition to Pagel’s lambda, we also tested for phylogenetic signal with Blomberg’s K [*phylosig()* function from the phytools R package (92)], and Moran’s I [*abouheif.moran()* function from the adephylo R package (93)].

**(vii) Correlations between top RL-ranked taxa and subregions in FCM fingerprint.** Variations in abundance of the top-ranked taxa with HNAcc were correlated with subregions in the FCM fingerprint (Fig. 5). A FCM fingerprint was constructed using the PhenoFlow package (58). In brief, a 128-by-128 binning grid was constructed for the green versus red fluorescence channels. A kernel density estimation was then applied (using a Gaussian kernel with a band width of 0.01) to retrieve cell densities per bin. Next, correlations between cell density and variation in taxa abundance were calculated using Kendall’s tau-b. This was done using the *cor()* function in R (v3.2).

**(viii) Validation of RL-selected taxa using Kendall’s tau-b and the Boruta algorithm.** Individual correlations between taxa and HNAcc or LNAcc were calculated using Kendall’s tau-b. This was done with the *kendalltau()* function in Scipy (v1.0.0). Another machine learning-based algorithm was used as well to associate taxa with HNAcc and LNAcc. The Boruta algorithm is a *wrapper* algorithm that makes use of Random Forests as a base classification or regression method in order to select all relevant variables in the function of a response variable (29). Similar to stability selection, the method uses an additional form of randomness in order to perform variable selection. Random Forests are fitted to the data multiple times. To remove the correlation to the response variable, each variable is assigned per iteration a so-called shadow variable, which is a permuted copy of the original variable. Next, the Random Forest algorithm is run with the extended set of variables, after which variable importances are calculated for both the original and shadow variables. The shadow variable that has the highest importance score is used as reference, and every variable with significantly lower importance, as determined by a Bonferroni corrected *t* test, is removed. Likewise, variables containing an importance score that is significantly higher are included in the final list of selected variables. This procedure can be repeated until all original variables are either discarded or included in the final set; variables that remain are given the label “tentative” (i.e., after all repetitions it is still not possible to either select or discard a certain variable). We used the boruta_py package to implement the Boruta algorithm (https://github.com/scikit-learn-contrib/boruta_py). Random Forests were implemented using *RandomForestRegressor()* function from scikit-learn (83), v0.19.1. Random Forests were run with 200 trees, the number of variables considered at every split of a decision tree was *p*/3, and the minimal number of samples per leaf was set at five. The latter were based on default values for Random Forests in a regression setting (94). The Boruta algorithm was run for 300 iterations, variables were selected or discarded at *P* < 0.05 after performing Bonferroni correction.

**Data availability.** All raw and processed data for this project are publicly available. The main GitHub repository for this project is https://deneflab.github.io/HNA_LNA_productivity/, which includes all of the processed data and the code for all figures, summary statistics, and Randomized Lasso regressions. The raw compressed 16S rRNA gene sequencing fastq files are available in the NCBI Sequence Read Archive under the following BioProject accession numbers: PRJNA414423 (inland lakes), PRJNA412983 (Lake Michigan), and PRJNA412984 (Muskegon Lake). The workflow for OTU generation can be found at the following GitHub repository: https://github.com/rprops/Mothur_oligo_batch. All flow cytometry data are available in the FlowRepository database (78): inland lakes (ID:FR-FCM-ZY9J), Lake Michigan, and Muskegon Lake (ID:FR-FCM-ZYZN). The supplemental information can be found at the following link: https://doi.org/10.6084/m9.figshare.8218775.v3.
